# Morphofunctional study of the tongue in the domestic duck (*Anas platyrhynchos f. domestica*, Anatidae): LM and SEM study

**DOI:** 10.1007/s00435-016-0302-2

**Published:** 2016-02-04

**Authors:** Kinga Skieresz-Szewczyk, Hanna Jackowiak

**Affiliations:** Department of Histology and Embryology, Poznan University of Life Sciences, Wojska Polskiego 71 C, 60-625 Poznań, Poland

**Keywords:** Feeding function, Tongue, Birds, Lingual glands, Mechanoreceptors

## Abstract

The domestic duck, as a representative of birds living in the water, is considered as a specialist filter-feeder. Behavioral observations of foraging revealed that these birds also use a terrestrial feeding mechanism such as grazing and pecking. This study examined the entirety of the lingual mucosa in relation to the structural adaptations required for this range of feeding activities. The structures on the lateral surfaces of the tongue, the conical and filiform papillae, constitute the food filtration apparatus. The process of pecking involves the spatula-shaped apex of the tongue and a specific horny plate—the lingual nail. In the grazing mechanism, large conical papillae and lamellae in the beak are required. Structures engaged in intra-oral transport include the median groove, lingual combs, the rostral border of the lingual prominence and distinct rows of conical papillae on the lingual prominence. Two types of keratinized epithelia, the ortho- and parakeratinized epithelium, as well as nonkeratinized epithelium cover individual areas of the tongue. The rostral and caudal lingual glands present in the lamina propria of the body, lingual prominence and root of the tongue produce mucus. The specific arrangement of Grandry and Herbst corpuscles form so-called bill-tongue organ monitoring food transportation. Our research confirm that the lingual mucosa in domestic duck is characterized by microstructural species-specific modifications of particular areas of the tongue, which is formed not only under the influence of the filtering mechanism, but also by terrestrial feeding mechanisms such as grazing or pecking.

## Introduction

The morphological structure of the tongue in birds is characterized by an abundance of structures resulting from a number of factors such as taxonomic affiliation, type of food intake, method of diet collection and the birds’ occupied environment. Harrison (1964) identified three groups of tongues with specialist structural adaptations to enhance the performance of their functions. The first tongue group are those used to capture and intake food utilizing a highly developed hyoid apparatus. The second functional group comprises of tongues with numerous, stiff papillae on the dorsal surface, adapted to holding and/or manipulating food. The third functional group is composed of tongues which are organs employed for retaining food in the oral cavity prior to swallowing.

The process of feeding in vertebrates is complex and generally is distinguished three stages: ingestion, intra-oral transport and swallowing (Schwenk 1989).

The behavioral observations of feeding mechanism revealed the presence of phylogenetic different between paleognathous and neognathous birds (Tomlinson 2000). In paleognathous birds, feeding behavior is based on the catch and throw mechanism, described as cranioinertial mechanism in which food is moved directly into the esophagus, without using the tongue. The neognathous birds use lingual feeding mechanism related to the complex movements of the beak and hyolingual apparatus. Sometimes neognathous birds use catch and throw mechanism, but it is used only during ingestion of large food particles and still requires complex movements of hyolingual apparatus. The exception among neognathous birds is toucan, hornbills and southern cassowary in which develops the so-called ballistic transport (Baussart et al. 2009; Baussart and Bels 2011; Harte et al. 2012).

Among neognathous birds, the Anseriformes are characterized by morphological specialization of tongue and beak which are involved in as many as three mechanisms of feeding, such as grazing, pecking and filtering food from water, and two types of transport called the under tongue transport and over tongue transport (Kooloos 1986; Kooloos et al. 1989; Van der Leeuw et al. 2003; Bels and Baussart 2006). In the order of Anseriformes, two subfamilies can be distinguished: Anserinae and Anatidae. The morphological structure of the tongue and its functions in Anserinae have been described for example in goose (Iwasaki et al. 1997; Jackowiak et al. 2011). The tongue morphology in Anatidae subfamily has not been previously described in detail. Wild duck (*Anas platyrhynchos)* is considered to be specialist filter-feeders, and the filtration mechanism is the main method of feeding (Van der Leeuw et al. 2003), yet they are also terrestrial feeders. The domestic duck (*Anas platyrhynchos f. domestica)*, domesticated form of wild duck, is an important food source, popular household pet and also laboratory model of Anatidae for experimental studies. Understanding its ability to intake particular foods and eating habits is an increasingly vital factor in rearing this animal, and therefore understanding how it processes this food is of paramount importance.

The hypothesis of this study is that feeding mechanisms of the domestic duck, typical for both aquatic and terrestrial life style, influenced on numerous structural adaptations of lingual mucosa. To verify this hypothesis, detailed observations were made on the morphology of the tongue in domestic ducks, with particular emphasis on macro- and microstructures of the lingual mucosa including the lingual papillae, lingual glands and mucosal epithelium in specific areas of the tongue.

## Materials and methods

The study was conducted on eight tongues of adult female domestic ducks (aged 6 months, average weight 3.5 kg) collected from a local slaughterhouse. The study was conducted in accordance with the guidelines set out by the Ethics Commission at the Poznan University of Life Sciences, and the national guidelines, Poland.

Immediately after slaughter, tongues were rinsed in saline and immersed in 10 % neutralized formalin. After a 24-hour fixation period, macroscopic photographic documentation was made using a digital camera.

In order to perform light microscopy and scanning electron microscopy (SEM) analysis, tissue samples were collected from the apex, body, lingual prominence, root and mechanical papillae from each tongue.

Tissue samples for light microscopy studies were dehydrated in a series of increasing concentrations of ethanol (70–96 %) and routinely embedded in Paraplast ^®^. Paraplast blocks were cut into sections of 4.5–5 µm in thickness. Tissue sections were stained using the Masson-Goldner trichrome histological staining technique (Romeis, 1989). Observations of the histological sections were performed using an Axioscope2plus light microscope (Zeiss, Germany). Photomicrographs were utilized on 10 histological sections. On each histological section, three measurements were made in order to determine 30 measurements of the height of the epithelium and its keratinized layer, using a Multiscan computer morphometric system (ver. 10.2, CSS, Warsaw, Poland).

Tissue samples undergoing SEM analysis were dehydrated in increasing concentrations of ethanol (70–96 %) and acetone (100 %). The samples were dried at the critical point using CO_2_ (Critical Point Dryer EM CPD300, Leica, Germany), mounted on aluminum tables covered with carbon tabs and coated with a gold layer measuring 15–30 nm in thickness (Gold Sputter S 150B, Edwards, England). Observations and photographic documentation were performed under a ZEISS 435 VP scanning electron microscope, at an accelerating voltage of 10-15 kV. On eight tissue samples, three measurements were made in order to determine a total of 24 measurements of the height and width of mechanical papillae, using a Multiscan computer morphometric system (ver. 10.2, CSS, Warsaw, Poland).

Histological measurements were statistically analyzed using Statistica (ver. 12.5, StatSoft, Poland) software. For each morphological feature, the following parameters were calculated: the mean value (*X*) with standard deviation (SD), the minimum value (min) and the maximum value (max).

## Results

### Macroscopic observations

**Fig. 1:**
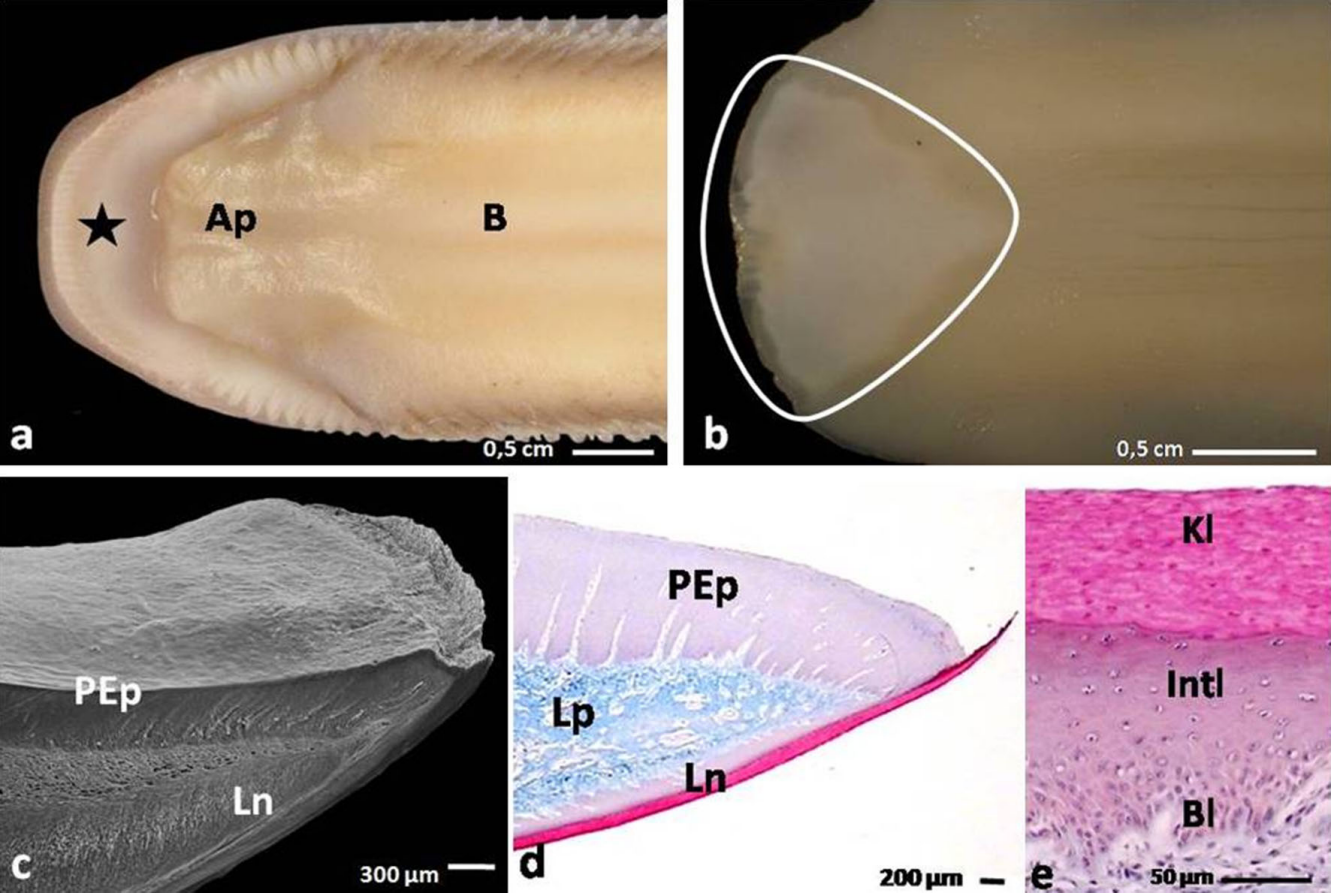
**a** Dorsal view on the rostral part of the tongue and the beak in the domestic duck. *Asterisk* shows the free tip of the beak. *A* apex of the tongue; *B* body of the tongue. **b** Ventral view on the apex of the tongue. *Continous line* marks the *triangular shape* of the lingual nail. **c** Dorsal view on the apex of the tongue with lingual nail protruding to the side of the apex. *PEp* parakeratinized epithelium on the dorsal surface of the apex; *Ln* lingual nail; SEM. **d** Sagittal cross section through the apex of the tongue. *PEp* parakeratinized epithelium on the dorsal surface of the apex; *Ln* lingual nail; *Lp* lamina propria; LM. **e** Cross section through the orthokeratinized epithelium of the lingual nail. *Bl* basal layer; *Int* intermediate layer; *Kl* keratinized layer; LM

**Fig. 2:**
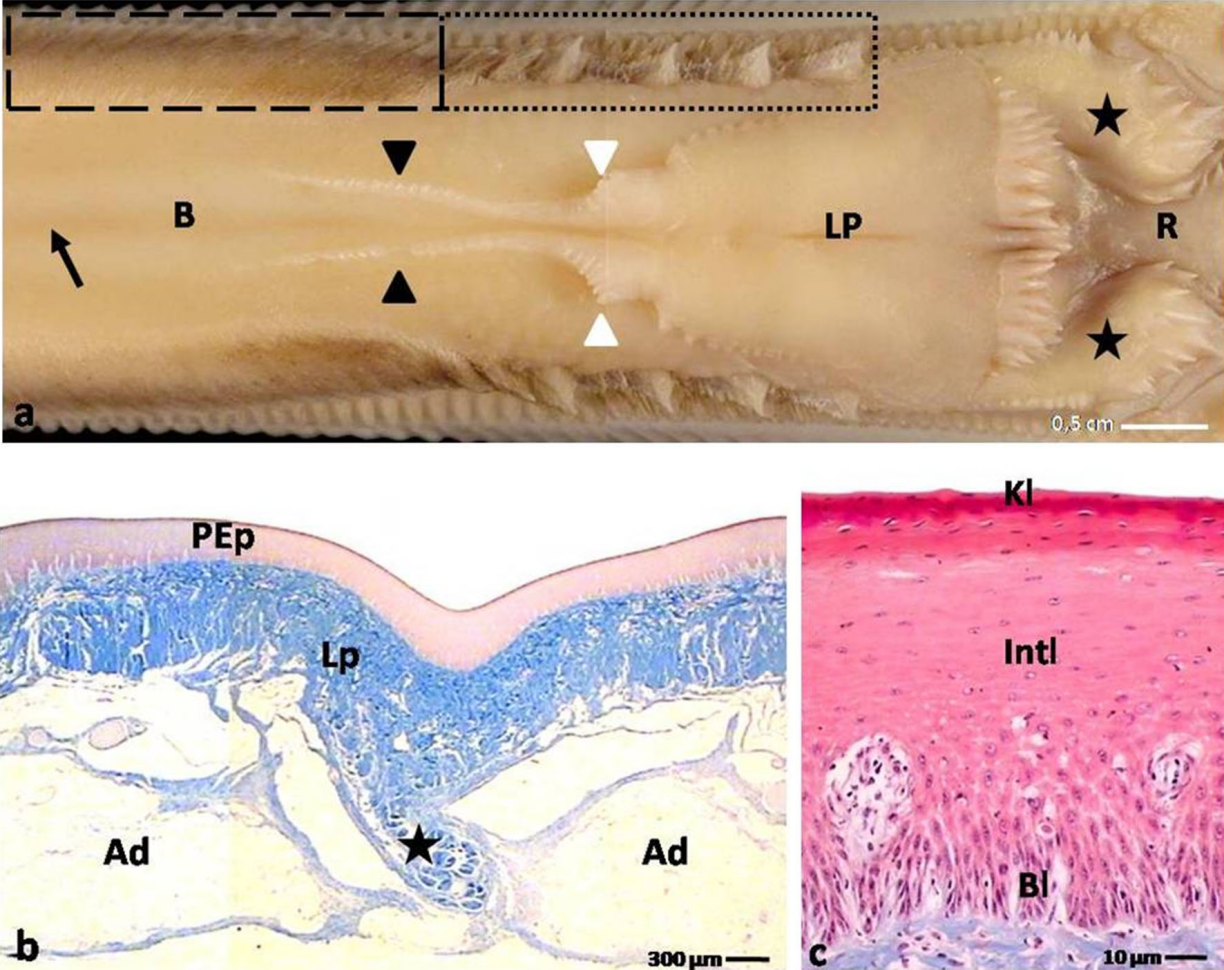
**a** Dorsal view on the body of the tongue and lingual prominence in the domestic duck. *Dashed line* shows small conical papillae. *Dotted line* points the large conical papillae. *Black arrows* show median groove of the body. *Black arrowheads* point the lingual comb. *White arrowheads* show turned up lingual comb. *Asterisk* point papillae on the lateral sides of the root. *B* body of the tongue; *LP* lingual prominence; *R* root of the tongue. **b** Cross section through the body. *Asterisk* shows connective tissue septum. *Ad* adipose tissue; *PEp* parakeratinized epithelium; *Lp* lamina propria; LM. **c** Cross section through the parakeratinized epithelium on the body. *Bl* basal layer; *Intl* intermediate layer; *Kl* keratinized layer; LM

The domestic duck tongue comprised of the apex, the body with the lingual prominence and the root (Figs. 1a, 2a). Tongues were attached to the bottom part of the bill by the frenulum. The tongue strictly occupied the oral cavity with the exception of the free tip of the rostral part of the bill (Fig. 1a).

The tongue in the domestic duck was narrow and elongated (Figs. 1a, 2a). The total length of the tongue averaged 6.3 cm, of which the apex averaged 0.8 cm in length, the body with the lingual prominence was 4.9 cm, and the root had a mean length of 0.6 cm. The average width of the tongue was 1.6 cm on the apex, 1.7 cm on the body, varied between 0.8 and 1.8 cm on the lingual prominence and 0.5 cm on the root.

#### The apex of the tongue

The apex of the domestic duck tongue was spatula-shape, and its dorsal surface presented as smooth and free of lingual papillae (Fig. 1a). On the ventral surface of the apex, there was a flat, triangular, white plate of the lingual nail and the edges of the structure stood out to the front and sides (Fig. 1b, c). The average length and width of the lingual nail through the middle was 1.3 and 1 cm, sequentially.

#### The body of the tongue

The dorsal surfaces of the tongue bodies were divided into two symmetrical parts by the shallow median groove (Fig. 2a). In the caudal part of the body, symmetrically on the sides of the median groove, two elevations of the mucosa were observed, which formed the left and right lingual combs with jagged edges (Figs. 2a, 4b). In front of the lingual prominence, the lingual comb turned up and subsequently merged with the rostral edges of the lingual prominence (Figs. 2a, 4b).

**Fig. 3:**
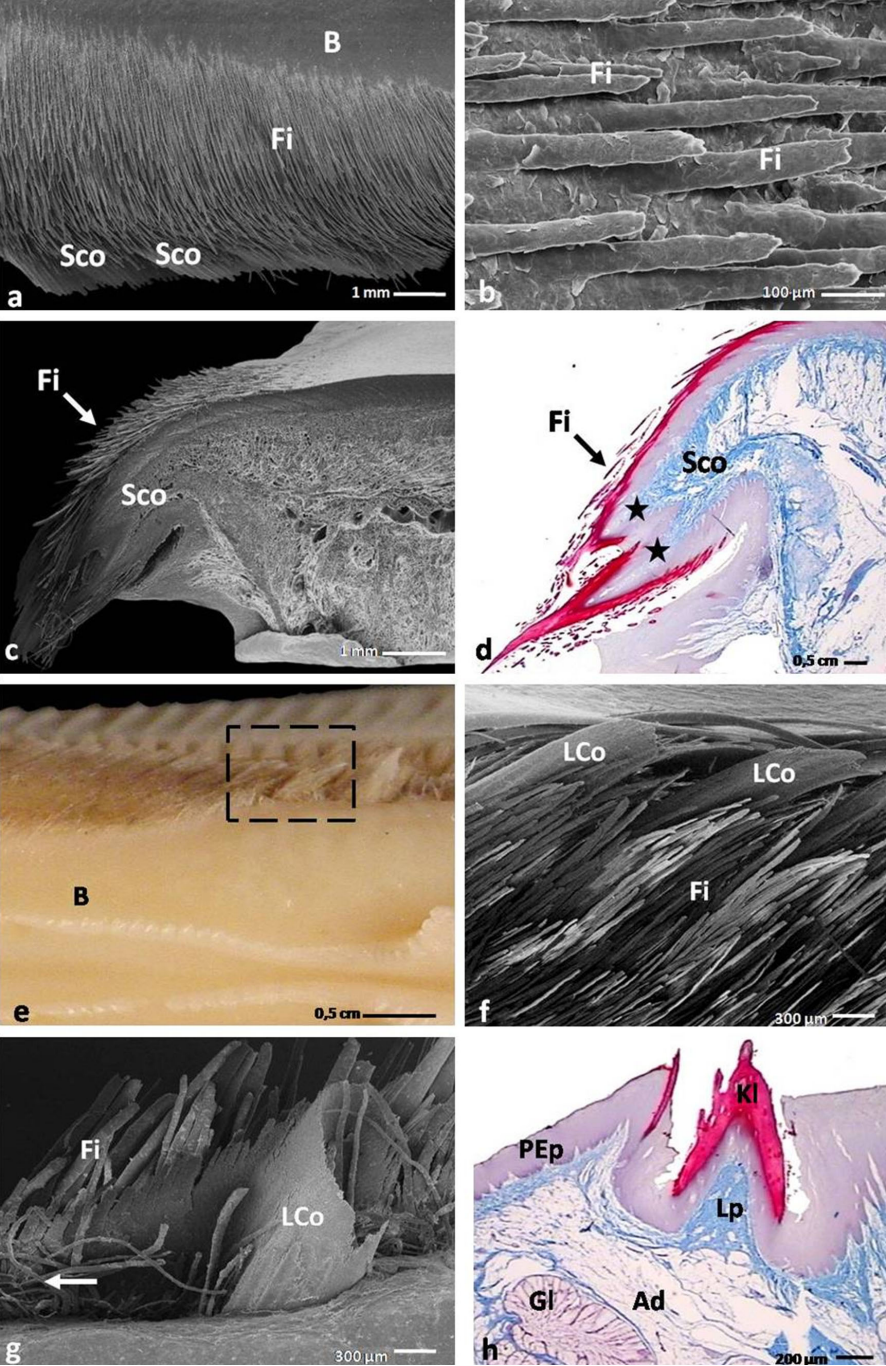
**a** Dorsal view on the dorso-lateral surface of the body of the tongue in the domestic duck. *B* body of the tongue; *Fi*, filiform papillae; *Sco*, small conical papillae; SEM. **b** Magnification of the filiform papillae, as keratinized processes of the epithelium. *Fi* filiform papillae; SEM. **c** Magnification of the small conical papillae covered with the brush of filiform papillae. *Fi* filiform papillae; *Sco* small conical papillae; SEM. **d** Cross section through the small conical papillae. *Asterisks* show ventral and dorsal connective tissue cores. *Fi* filiform papillae; *Sco* small conical papillae; LM. **e** Dorsal view on the dorso-lateral part of the body of the tongue in the domestic duck. *Dashed line* points the two large conical papillae in the caudal part of the lingual body. *B* body of the tongue. **f** Magnification of the two conical papillae with frayed tips. *Fi* filiform papillae; *Lco* large conical papillae; SEM. **g** Magnification of the large conical papillae in shape of a fountain pen. *Arrow* shows twisted processes of the filiform papillae. *Fi* filiform papillae; *Lco* large conical papillae; SEM. **h** Cross section of the large conical papillae. *Ad* adipose tissue; *Gl* rostral lingual glands; *Lp* lamina propria; *Kl* keratinized layer of the orthokeratinized epithelium; *PEp*, parakeratinized epithelium; LM

Symmetrically, along both edges of the body, there were three types of mechanical papillae–large and small conical papillae and filiform papillae (Figs. 2a, 3a, e, f, g). On the smooth lateral surfaces of the body of the tongue, 16–18 openings of the rostral lingual glands were linearly arranged. The average distance between openings was between 0.9 and 1.8 mm.

#### Small conical papillae of the body

In the rostral part of the body, 14 pairs of the small conical papillae were observed. Each papilla had the shape of a flattened plate with jagged ends (Fig. 3a). The papillae were directed toward the bottom of the tongue at an angle of 40–45°.

#### Large conical papillae of the body

In the caudal part of the body, six pairs of large conical papillae of different shapes were present directly behind the small conical papillae. The first four pairs of these papillae were found in the form of slightly flattened cones with a caudal concave surface resembling the shape of the nib of a fountain pen (Fig. 3g). Two other pairs of large conical papillae took the form of cones with frayed tips (Fig. 3e, f). These papillae lay directed caudally to the root of the tongue and were arranged at an angle of 20–30° to the lingual body.

#### Filiform papillae of the body

Filiform papillae in the rostral part of the lingual body formed a dense covering overlapping small conical papillae, which were located underneath the filiform papillae (Fig. 3a, c). The filiform papillae on the caudal part of the body presented on the medial side of the large conical papillae and formed twisted processes (Fig. 3g), while filiform papillae between large conical papillae formed densely arranged, simply structured long processes (Fig. 3f, g).

#### The lingual prominence

The lingual prominence had the shape of a triangle, the base of which was directed toward the root of the tongue (Fig. 2a). The lingual prominence was divided into two symmetrical parts by a slight median groove (Fig. 5a). The rostral serrated edges of the prominence raised above the lingual body (Fig. 5a). On the caudal edge of the prominence, rows of conical papillae had formed (Fig. 5a). On the caudo-lateral surfaces of the prominence, there were 2–3 openings of the caudo-lateral lingual glands.

**Fig. 4:**
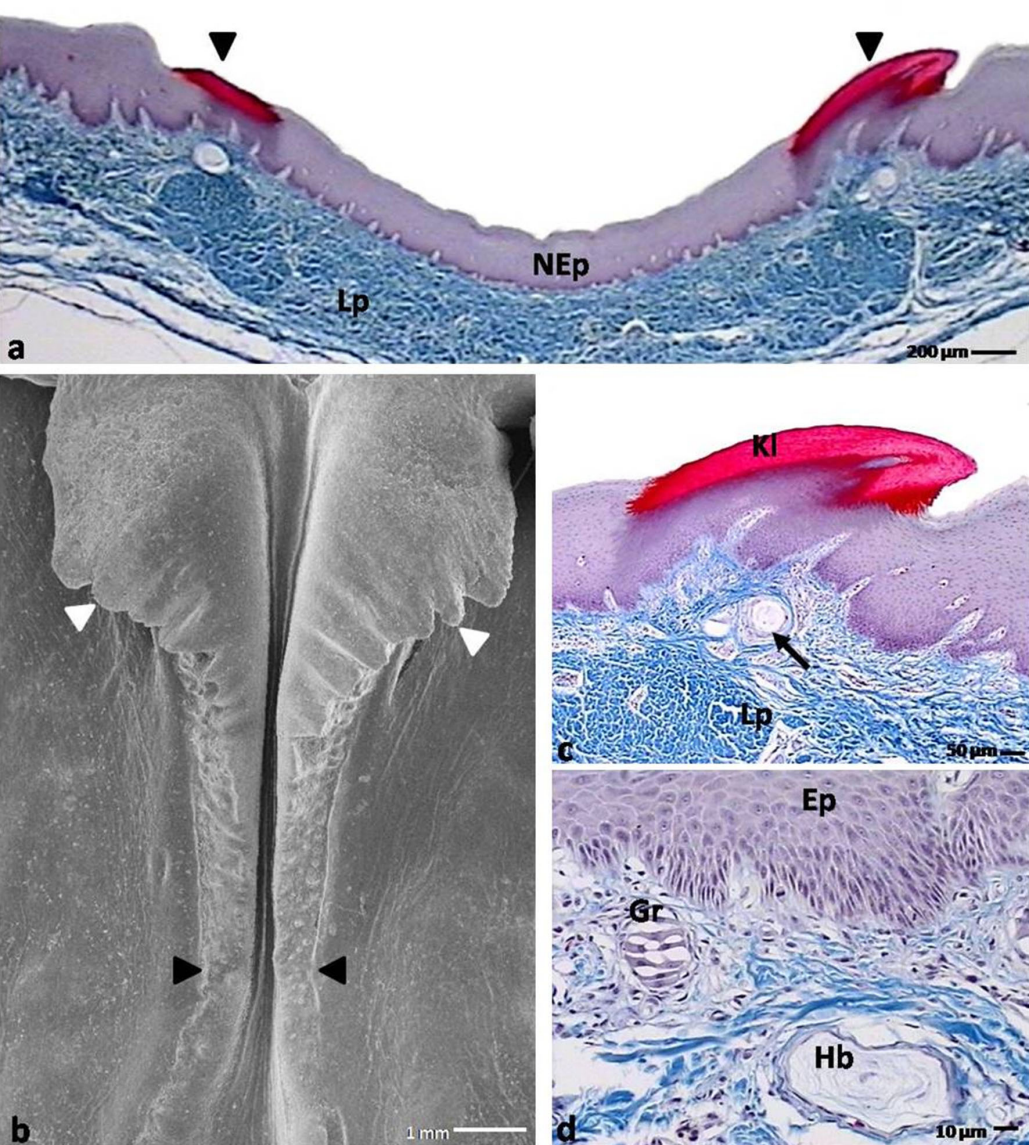
**a** Cross section through the caudo-median part of the lingual body in the domestic duck. *Arrowheads* point the* right* and* left* lingual combs of the mucosa. *NEp* nonkeratinized epithelium; *Lp* lamina propria; LM. **b** Magnification of the caudo-median part of the body. *Black arrowheads* show right and left lingual combs. *White arrowheads* point the serrated turned up lingual combs; SEM. **c** Cross section through the right lingual comb in the domestic duck. *Arrow* points the Herbst corpuscle. *Lp* lamina propria; *Kl* keratinized layer of the orthokeratinized epithelium; LM. **d** Magnification of the mechanoreceptors beneath the lingual comb. Ep. epithelium; *Gr* Grandry corpuscle; *Hb* Herbst corpuscle; LM

**Fig. 5:**
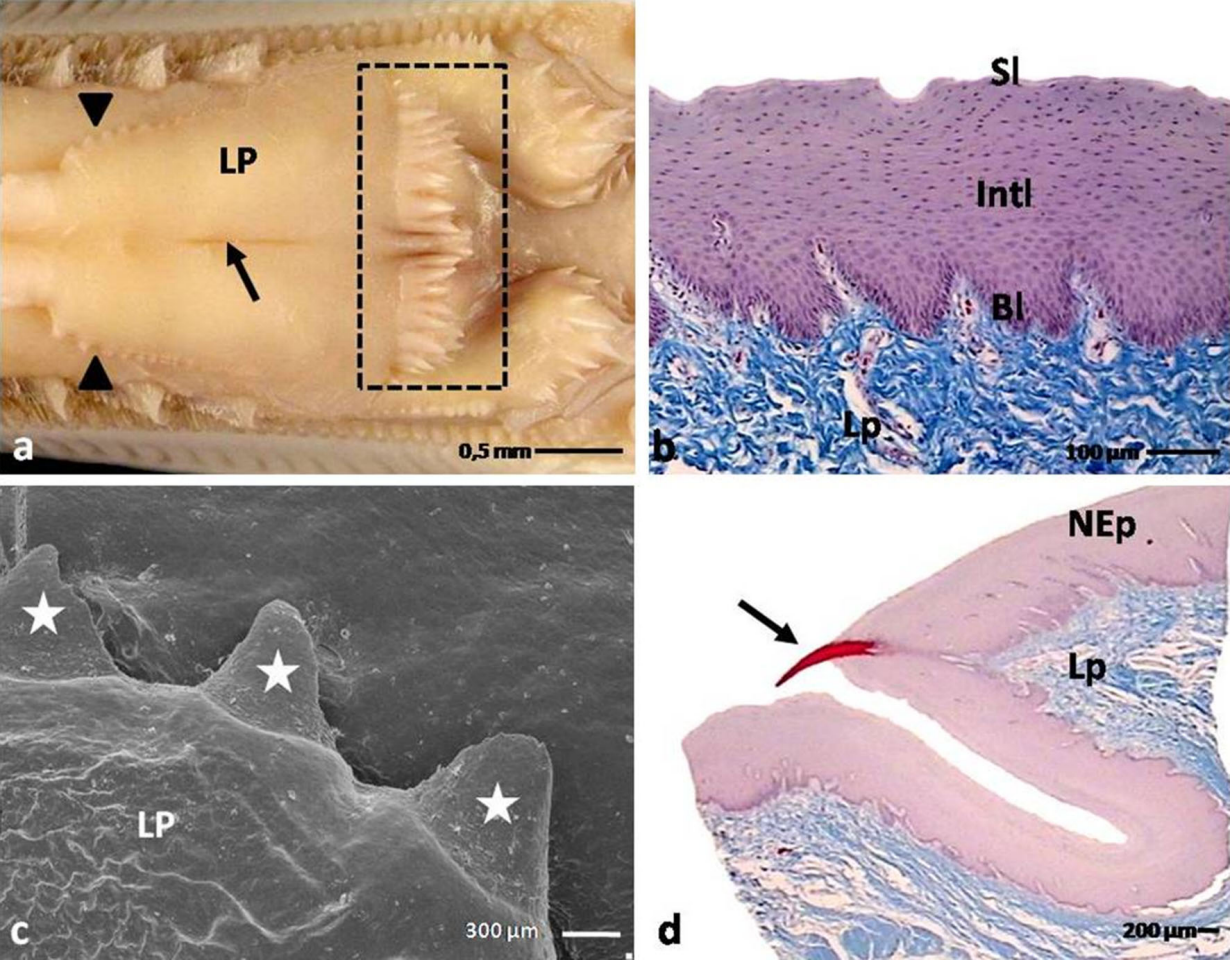
**a** Dorsal view on the surface of the lingual prominence and the root of the tongue in the domestic duck. *Dashed line* points rows of conical papillae on the caudal border of the lingual prominence. *Arrow* shows median groove. *Arrowheads* show serrated rostral part of the lingual prominence. *LP* lingual prominence; *R*, root of the tongue. **b** Cross section through the nonkeratinized epithelium of the lingual prominence. *Bl* basal layer; *Int* intermediate layer; *Sl* superficial layer; *Lp* lamina propria; LM. **c** Dorsal view on the border of the rostral part of the lingual prominence. *Asterisks* point serration. *LP* lingual prominence; SEM. **d** Cross section through the rostral part of the lingual prominence with keratinized processes (*arrow*). *NEp* nonkeratinized epithelium; *Lp* lamina propria; LM

#### Conical papillae of the lingual prominence

**Fig. 6:**
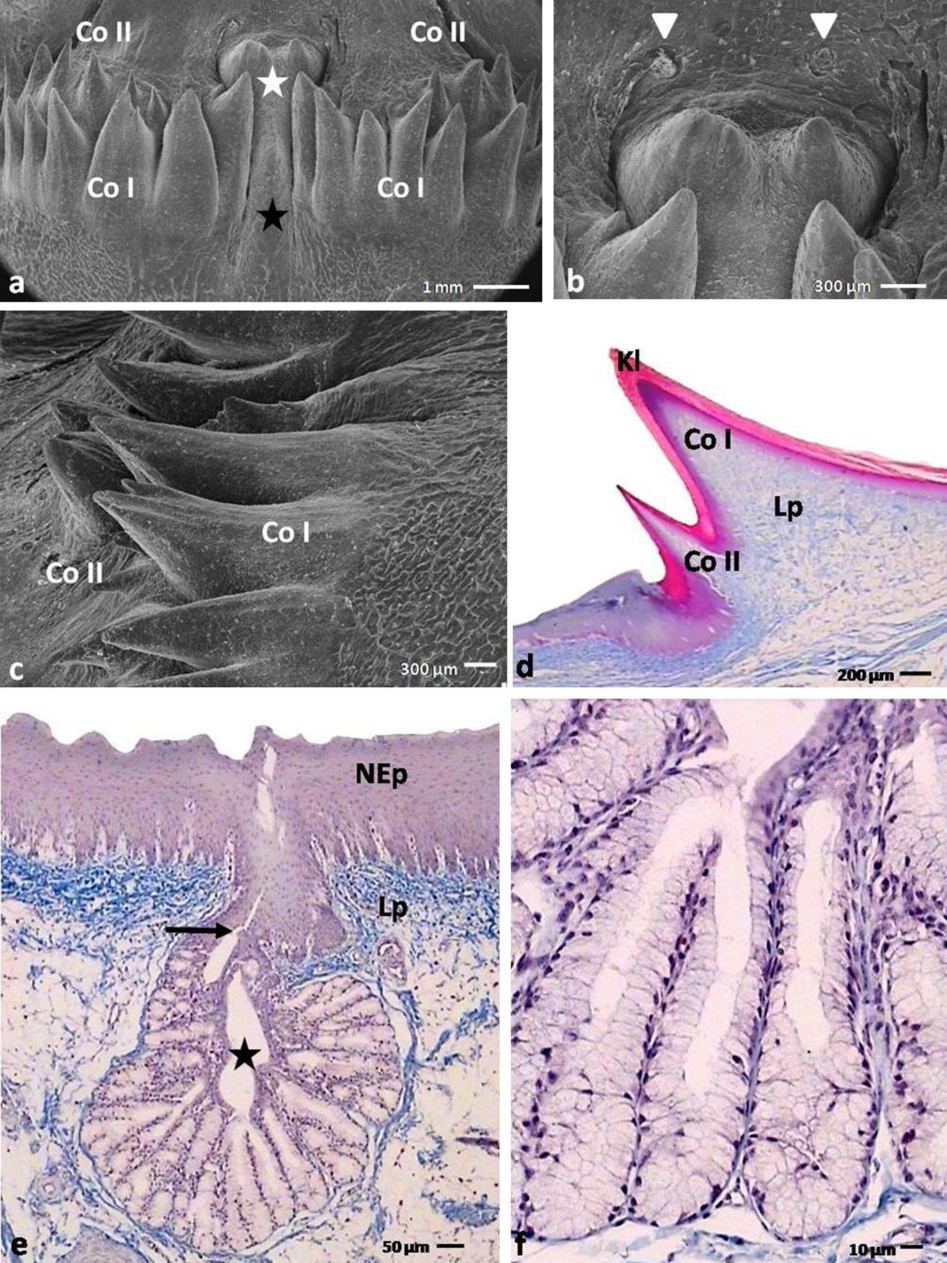
**a** Dorsal view on the caudal part of the lingual prominence in the domestic duck. *Black asterisk* shows median elevation of the mucosa. *White asterisk* points two conical papillae with a common base. *Co I* conical papillae in the first row; *Co II* conical papillae in the second row; SEM. **b** Magnification of the surface of the root behind conical papillae of the lingual prominence. *Arrowheads* point openings of the caudo-median lingual glands; SEM. **c** Lateral view on the caudally pointed conical papillae. *Co I* conical papillae in the firs row; *Co II* conical papillae in the second row; SEM. **d** Cross section through the conical papillae of the lingual prominence. *Co I* conical papillae in the firs row; *Co II* conical papillae in the second row; *Kl* keratinized layer of the orthokeratinized epithelium; *Lp* lamina propria; LM. **e** Cross section through the caudo-median lingual glands in the root of the tongue. *Asterisk* points the wide collecting chamber. *Arrows* shows short secretory duct. *Ad* adipose tissue; *Lp* lamina propria; LM. **f** Magnification of the caudo-median lingual glands arranged in lobules; LM

The conical papillae of the lingual prominence were arranged in two rows directed obliquely and caudally (Fig. 6a). Additionally, papillae in the first and second rows were divided into two left and right groups, in the midline of the prominence a distinct mucosa elevation was observed with its base located at the second rows of papillae (Fig. 6a).

In the first row, 16 conical papillae were observed, with 8 papillae on each of the right- and left-hand sides of the prominence. Similarly, in the second row there were 12 conical papillae, with 6 papillae on each side. The tips of the conical papillae of the lingual prominence were pointed and bent over the flat surface of the root (Fig. 6c).

#### The root of the tongue

The area of the root tongue, adjacent to the laryngeal prominence, was the smallest part of the tongue. Its surface was located below the lingual prominence (Fig. 2a). On both sides of the root, two round papillae with smaller spinal processes were detected (Fig. 2a). In the median part of the root, three pairs of the glandular openings of the caudo-median lingual glands arranged linearly were observed (Fig. 6b).

### Microscopic observations

The mucosa of the tongue in the domestic duck consisted of a multilayered epithelium that covered the connective tissue lamina propria. The mechanoreceptors and the mucous glands were structures observed subepithelially.

A characteristic feature of the tongue in the domestic duck was the presence of the yellow adipose tissue under the lamina propria of the mucosa on the body, lingual prominence and the root of the tongue (Figs. 2b, 3h). The adipose tissue covered the internal skeleton of the tongue formed by the elongated entoglossum cartilage of the hyoid apparatus. The yellow adipose tissue was particularly well developed in the caudal part of the body of the tongue and on the lingual prominence, taking the shape of a cushion. In the rostral part of the body, the fat tissue was divided into two parts, right and left bands, by a thin vertical connective septum (Fig. 2b). In the caudal part of the body, at the location of the lingual comb, the yellow adipose tissue formed a single band. The adipose tissue surrounded the entire complex of the lingual glands (Fig. 3h).

#### Epithelia of the lingual mucosa

Observations of the cross sections in all areas of the tongue mucosa in the domestic duck showed that it was covered by a multilayered ortho- and parakeratinized epithelium and nonkeratinized epithelium.

*Orthokeratinized epithelium* was found on the ventral surface of the apex of the tongue, where it formed the lingual nail and was also present on the lingual comb and on the conical papillae of the body and lingual prominence (Figs. 1d, 3d, h, 4c, 6d). This epithelium was composed of basal, intermediate and keratinized layers (Fig. 1e). The basal layer consisted of elongated cells with elliptical cell nuclei. Masson-Goldner staining revealed a different coloration of the cell cytoplasm in the intermediate layer, dividing the layer into two zones. In the lower zone cell, morphology was polygonal with oval nuclei arranged horizontally with one or two nucleoli. The cytoplasm of these cells was only faintly dyed pink. The upper zone of the intermediate layer was built of strongly flattened cells, most of which lacked cell nuclei or, where present, had a flat nucleus. The cellular cytoplasm was intensely stained red. The cells in the keratinized layer were also heavily flattened and devoid of cell nuclei, and the cytoplasm was dyed red. The height of the orthokeratinized epithelium was 229.4 µm, and its keratinized layer was 76.2 µm thick (Table 1).

**Table 1:** Morphometry of the epithelium of the lingual mucosa in adult domestic duck

Part of the tongue	The height of the epithelium of the mucosa (μm) *X* (min.–max.) SD	The height of the keratinized layer of the mucosal epithelium (μm) *X* (min.–max.) SD
Apex of the tongue–dorsal surface	877.9 (748.4–1083.3) 118.3	14.3 (11.9–16.7) 1.5
Apex of the tongue–ventral surface	229.4 (215.6–247.5) 11.2	76.2 (73.8–79.5) 2.2
Body of the tongue	345.6 (334.4–354.9) 7.8	9.2 (7.4–11.5) 1.6
Lingual prominence	307.7 (272.6–341.0) 30.6	–
Root of the tongue	169.9 (125.0–207.7) 33.3	–

The parakeratinized epithelium was situated on the dorsal surface of the apex and the body of the tongue and was also assembled by basal, intermediate and keratinized layers (Figs. 1d, 2c). The basal and intermediate layers were morphologically comparable to those in the orthokeratinized epithelium. The structure of the keratinized layer was structurally varied, depending on the area of the tongue. The cells of the keratinized layer on the dorsal surface of the apex displayed only a partially flattened cell nucleus, and the cytoplasm was weakly colored in red, giving the impression of a discontinuous keratinized layer. The keratinized layer of the body of the tongue had flattened cell nuclei with highly condensed chromatin. The cytoplasm of these cells dyed intensely red and was visible on the tissue cross sections as a single, continuous layer. The height of the epithelium on the dorsal surface of the apex was 877.9 µm, and on the body it was 345.6 µm (Table 1). The average height of the keratinized layers on the apex and body was measured at 14.3 and 9.2 µm, respectively (Table 1).

The nonkeratinized multilayered epithelium covered the surface of the prominence and root of the tongue (Figs. 5b, 6a). The basal layer was histologically the same as described in the keratinized epithelium. The intermediate layer consisted of polygonal cells with oval cell nuclei and one or two nucleoli. The cytoplasm of these cells, following Masson-Goldner staining, was observed as taking up the pink dye in a uniform manner. The cells in the superficial layer were flat, the cell nuclei were heavily flattened, and cell cytoplasm was evenly stained pale pink. The average height of the epithelium on the lingual prominence was 307.7 µm, and on the root of the tongue reached only 169.9 µm (Table 1).

#### Mechanical papillae of the tongue and the lingual comb

The small conical papillae situated on the lateral borders of the lingual body composed of double, dorsal and ventral connective tissue cores, directed toward the ventral surface of the tongue (Figs. 3c, d). The ventral connective tissue core was longer and covered by a shorter dorsal connective tissue core of papilla. The average length of the dorsal connective tissue core was 2002.2 µm, and the average length of the ventral connective tissue core was 2600.1 µm (Table 2). Both connective tissue cores of the small conical papillae reached average width of 975.7 µm (Table 2). The small conical papillae were covered with an orthokeratinized epithelium with an average height of 169.3 µm, while the thickness of the keratinized layer was 82.8 µm (Table 2).

**Table 2:** Morphometry of the mechanical papillae in adult domestic duck

Type of the mechanical papillae		The height of the papillae (μm) *X* (min.–max.) SD	The width of the papillae (μm) *X* (min.–max.) SD	The height of epithelium (μm) *X* (min.–max.) SD	The height of the keratinized layer (μm) *X* (min.–max.) SD
Small conical papillae on body of the tongue					
Dorsal tissue core		2002.2 (1749.2–2322.2) 200.9	975.7 (879.3–1034.8) 64.4	169.6 (153.2–188.3) 15.5	82.8 (67.6–105.1) 11.6
Ventral tissue core		2600.1 (2415.0–2755.2) 147.8			
Large conical papillae on body of the tongue		2773.7 (2486.1–2925.7) 207.5	1553.1 (1516.0–1632.3) 53.8	327.8 (264.8–387.7) 49.0	155.6 (103.3–232.4) 36.6
Conical papillae of the lingual prominence					
I row		1800.8 (1534.9–2277.6) 266.7	678.2 (507.2–1099.7) 229.4	162.9 (116.0–258.9) 61.7	82.4 (39.4–127.9) 34.5
II row		709.2 (476.9–930.9) 151.4	327.6 (237.8–436.1) 68.8	65.7 (49.7–90.4) 14.5	35.1 (27.4–38.4) 3.5
Filiform papillae on body of the tongue		1513.4 (1210.8–1715.1) 170.1	45.8 (34.4–61.3) 8.8	–	–

Each large conical papilla of the caudo-lateral part of the body of the tongue in the domestic duck had a single connective tissue core coated with a multilayered orthokeratinized epithelium (Fig. 3h). The average length of the papillae was 2773.7 µm, and the width was 1553.1 µm (Table 2). The height of the epithelium of the large conical papillae was 327.8 µm, and the height of the keratinized layer was 155.6 µm (Table 2).

The filiform papillae of the body did not have connective tissue cores and were composed of keratinized processes of the orthokeratinized epithelium (Fig. 3b, d). The filiform papillae reached an average length of 1513.4 µm and an average width of 45.8 µm (Table 2).

The conical papillae of the lingual prominence comprised of single connective tissue cores covered with a multilayered orthokeratinized epithelium (Fig. 6d). The conical papillae in the first row were larger and their average length was calculated as 1800.8 µm and the width at the base was 678.2 µm (Table 2). The papillae in the second row are shorter and narrower. The length of papillae in the second row averaged 709.2 µm, and the width at the base was between 327.6 µm (Table 2). The height of the epithelium of the conical papillae in the first row was 162.9 µm, half of which was the keratinized layer of 82.4 µm in height (Table 2). The conical papillae in the second row were covered with a lower epithelium, with a height of 65.7 µm, while the height of the keratinized layer was 35.1 µm (Table 2).

The lingual comb was assembled from thin, triangular connective tissue cores covered with multilayered orthokeratinized epithelium (Fig. 4a, c). The epithelium of the sulcus between the right and left comb presented as a multilayered nonkeratinized epithelium (Fig. 4a).

The rostral edge of the lingual prominence was covered with a multilayered nonkeratinized epithelium. A characteristic feature of this part of the lingual prominence in the domestic duck was serration (Fig. 5c). Figure 5d shows that the serrations were keratinized processes with small connective tissue cores.

#### Lingual glands

Rostral and caudal lingual glands were found in the lamina propria of the mucosa within the domestic duck. The glands presented as complex, tubular glands secreting mucus. The secretory units of the rostral and caudal lingual glands were arranged in lobules surrounded by a thin band of loose connective tissue and externally encapsulated by yellow adipose tissue (Figs. 3h, 6f). The glands were characterized by a wide collecting chamber and a short excretory duct (Fig. 6e). The rostral lingual glands were located along both sides of the entoglossum cartilage in the caudal part of the body and the rostral part of the lingual prominence. The caudal lingual glands existed in two groups: the caudo-lateral glands located on the sides of the caudal part of the lingual prominence and the root of the tongue, and the caudo-median glands extant under the epithelium in the central part of the root.

#### Mechanoreceptors

In the lamina propria of the lingual mucosa, two types of mechanoreceptors were present, the Herbst and Grandry corpuscles. Both types of these sensory corpuscles were present subepithelially on the apex of the tongue, on the periphery of the lingual nail, under the lingual comb, in the connective tissue cores of the conical papillae of the body and under the rostral border of the lingual prominence (Fig. 4c).

The Herbst corpuscles were elliptical in shape and were composed of concentric lamellae (Fig. 4d). The center of the corpuscles contained the end of a nerve fiber. The Grandry corpuscles were made up of 2–6 flat cells, stacked to form a sandwich-like structure (Fig. 4d). On the histological sections, a characteristic arrangement of those corpuscles to each other was observed. The Grandry corpuscles were located in closer proximity to the epithelium than the Herbst corpuscles (Fig. 4d). Morphometric studies showed that the Herbst corpuscles varied in diameter dependent on their location. The corpuscles beneath the small conical papillae were 88.8 µm in diameter, and on the edge of the lingual prominence they were 145.4 µm. In contrast, the diameter of the Grandry corpuscles was on average 29.5 µm, which did not differ greatly upon location.

## Discussion

Literature dealing with the feeding behavior in wild birds shows that Anseriformes were distinguished by three ways of gathering food: pecking, grazing and filter-feeding (Van der Leeuw et al. 2003; Baussart et al. 2009). These studies showed that, between Anserinae and Anatidae, there are also differences in the transportation of food into the esophagus.

After analyzing the three methods of feeding and the two types of transport, and on the basis of the conducted detailed macro- and microscopic observations of the tongue in the duck, it was possible to determine the functional adaptation of individual parts of the tongue.

The first type of food intake in Anatidae is pecking which starts with grabbing the grains by the front part of the beak. The main structure involves in this feeding behavior is the apex with the lingual nail. The lingual nail stands out to the front and side of the apex and can act as a spoon for lifting grains. Similar observations have been made by Jackowiak et al. (2011) in the domestic goose. Although the lingual nail is a hard keratinized structure, it is very flexible and efficient in collecting food (Homberger and Brush 1986). Microscopic observations of the cross section of the apex showed that in the mid-length of the apex it did not have an entoglossal cartilage and was built of loose connective tissue. The lingual nail, which comprised of the orthokeratinized epithelium with a thick keratinized layer, may play an important role as the external skeleton supporting the apex of the tongue. This statement is supported by the results of morphometric measurements, which showed that the keratinized layer is up to one-third of the height of the epithelium.

The second type of food intake in Anatidae is grazing. The wild duck uses the lateral rims of the beak to grab the leaves of grass, which are then broken off and blades of grass are hold by pressing the lingual prominence to the palate (Van der Leeuw et al. 2003). The morphological structures directly linked to grazing in the domestic duck are the large conical papillae. They have shape of cones directed to the root of the tongue and are located at the latero-caudal part of the lingual body. They are compatible to the lamellae in bottom part of the beak and act like scissors. The small conical papillae have a shape of plate directed to the bottom of the tongue and do not take part in the grazing. Comparing current data with observations made in the domestic goose (Jackowiak et al. 2011), we can state the tongue in the domestic duck is less well adapted for cutting grass, because only the conical papillae in the caudal part of the body of the tongue are involved in this action. What may be due to the fact that grazing is not the main mechanism of feeding.

The unique type of food ingestion in Anatidae is filter-feeding. Behavioral studies performance by Kooloos et al. (1989) and Zweers et al. (1997) showed that the water is pumped into the oral cavity when the beak is open, the tongue is retracted, and the lingual body is raised. When the beak is closing, the tongue is retracted and the lingual body is depressed, the water and food are forced to move on the dorsal surface of the tongue, just before lingual prominence. During another retraction of the tongue, the lingual body is raised what causes that the water with the food samples is moved on the lateral sides of the lingual prominence. The water is then removed outside. The current research demonstrates that the first barrier stopping large items of food is the serrated edge of the lingual prominence. The second barrier is the so-called filtering apparatus, which is formed by small and large conical papillae of the body and the filiform papillae. Based on observations, it appears that the effectiveness of filtration for large conical papillae in the domestic duck is smaller compared to the small conical papillae, due to the shape of the papillae, their caudal orientation and a less dense arrangement of the filiform papillae. The filiform papillae in the rostral part of the body can act as a brush retaining even the smallest food items, which is adapted as a dense filtering apparatus, efficiently stocking finer particles as compared to those structures in the goose (Jackowiak et al. 2011).

In the wild duck has been preserved catch and throw transport of grains, diameter of which is smaller than that of a pea, and is also utilized to move grass blades (Kooloos 1986; Zweers et al. 1997; Tomlinson 2000; Van der Leeuw et al. 2003). These birds feed mainly on food immersed in water by using the filter-feeding mechanism (Kooloos 1986; Zweers et al. 1997; Van der Leeuw et al. 2003). During filtration, duck use typical for neognathous bird, lingual feeding mechanism and under tongue transport (Tomlinson 2000; Van der Leeuw et al. 2003). This method of food transport has decided about formation of the specific structures of the lingual mucosa. The present study revealed that mucosal structures involved in the transportation of food in the domestic duck are midline groove, which acts as a gutter in which food is transported, the lingual comb, which is engaged in the division of food particles into two parts, and raised serrated edges of the rostral part of the lingual prominence facilitate the under tongue transport. The conical papillae of the lingual prominence help in the transport of food into the esophagus, both during catch and throw transport and under tongue transport, while two papillae on the sides of the root may be used to re-direct food onto one track, forming a bite of food and protection from falling out from the oral cavity.

The current studies in the domestic duck have also shown that the lamina propria of the lingual comb, the edges of the body, and the rostral edges of the lingual prominence have two types of specifically arranged mechanoreceptors—the Grandry and Herbst corpuscles. An interesting feature was the mutual arrangement of these corpuscles. The Grandry corpuscles were generally positioned more subepithelial than the Herbst corpuscles. Leitner and Roumy (1974) found that in the skin of the beak and in the tongue in the domestic duck these corpuscles may be arranged on the same level. The Herbst and Grandry sensory corpuscles differ in terms of their functions. According to literature sources, the Herbst corpuscles are responsible for the reception of mechanical stimulation, mainly vibration (Gottschaldt and Lausmann 1974). In turn, the Grandry corpuscles, due to the similar structure to the Merkel cells, are attributed to the function of slow acting mechanoreceptors (Halata and Grim 1993; Toyoshima 1993, Kumamoto et al. 1995; Halata et al. 2003). The Herbst and Grandry corpuscles are found in both the beak and oral cavity in the domestic duck; however, mainly they are distributed in the caudal part of the beak (Leitner and Roumy 1974). The emu and ostrich are equipped only with the Herbst corpuscles (Crole and Soley 2014). Researchers have determined that skin mechanoreceptors in the rostral part of the beak and in the oral cavity form the so-called bill tip organ (Gottschaldt and Lausmann 1974; Berkhoudt 1980; Gentle and Breward 1986; Halata and Grim 1993). After analyzing the distribution of the sensory corpuscles in the skin of the beak and oral cavity, and comparing them with current results in the mucosa of the tongue, it may be stated that as previously stated in the domestic goose (Jackowiak et al. 2011), they all form together the so-called bill-tongue organ, which is responsible for receiving numerous mechanical impulses that originate during the exploration, ingestion and transportation of food.

Studies on the distribution and the structure of the epithelium covering the tongue in the domestic duck revealed that besides the type of food and its consistency, the methods of food collection and transport have a significant effect on the degree of keratinization of the epithelium (Skieresz-Szewczyk et al. 2014). A strongly keratinized epithelium, in this case the orthokeratinized epithelium, was found mainly on the mechanical papillae and the lingual comb, and it is likely that they are situated there because those parts are actively involved in grazing, filtering and transportation of food, and thus they are subject to stronger mechanical pressure. On the dorsal surface of the apex and the body of the domestic duck tongue, where food is moved to the esophagus, a very thick parakeratinized epithelium was observed. A lack of a protective, thick keratinized layer is potentially compensated for by a thickening of the epithelium, which undergoes renewal. The presence of the nonkeratinized epithelium on the lingual prominence and the root may be attributed to the fact that during the so-called under tongue transport and the catch and throw mechanism, those parts of the tongue have a less contact with collected food surrounded by mucus.

Lamina propria of the mucosa in the domestic duck is equipped with numerous complex anterior and posterior lingual glands, which are typical for other birds (Iwasaki and Kobayashi 1986; Kobayashi et al. 1998; Liman et al. 2001; Jackowiak and Godynicki 2005; Rossi et al. 2005; Emura et al. 2010a, b, 2011). The tongue is characterized by many openings located on the lateral surfaces of the body and the lingual prominence and on the dorsal surface of the root. Pattern of openings localization is typical for other Anseriformes (Jackowiak et al. 2011). Secretion of the lingual mucous glands is mainly used to wet the oropharynx and bind food particles. In the case of domestic duck, living mostly in the aquatic environment and collecting hydrated shoots of plants, the function of food and oral cavity humidification seems to be less important. It should be noted that during grazing and pecking of dry food they help to moisturize food particles and prepare them for transport into the esophagus.

The currently presented, detailed description of the macro- and microscopic structures of the tongue in the domestic duck pointed to a number of microstructural adaptations of the mucosa formed under the influence of different feeding mechanisms in comparison with other birds. The rich sculpture of the tongue in the domestic duck, expressed in a characteristic arrangement and structure of the mechanical papillae of the body, the presence of the lingual comb and a specific shape of the lingual prominence, points to adaptations to the active and efficient filtering of food from water as a main feeding mechanism of this water-living bird. Nevertheless, the tongue in the domestic duck is also adapted to performing typical terrestrial activities, including grazing and pecking, which is expressed in appropriately shaped conical papillae of the lingual body and the special structure, which is the lingual nail on the ventral surface of the apex. These investigations help us to understand not only the anatomy and histological features of the domestic duck tongue, but also understand avian adaptations to differing feeding mechanisms.
